# Adeno-associated virus-mediated expression of activated factor V (FVa) for hemophilia phenotypic correction

**DOI:** 10.3389/fmed.2022.880763

**Published:** 2022-08-05

**Authors:** Junjiang Sun, Xiaojing Chen, Zheng Chai, Hongqian Niu, Amanda L. Dobbins, Timothy C. Nichols, Chengwen Li

**Affiliations:** ^1^Division of Pharmacoengineering and Molecular Pharmaceutics, Eshelman School of Pharmacy, University of North Carolina at Chapel Hill, Chapel Hill, NC, United States; ^2^Gene Therapy Center, School of Medicine, University of North Carolina at Chapel Hill, Chapel Hill, NC, United States; ^3^Department of Pathology and Laboratory Medicine, University of North Carolina at Chapel Hill, Chapel Hill, NC, United States; ^4^Department of Pediatrics, University of North Carolina at Chapel Hill, Chapel Hill, NC, United States

**Keywords:** hemophilia, AAV (adeno-associated virus), gene therapy, activated factor V, inhibitor

## Abstract

Adeno-associated virus (AAV) gene therapy has been successfully applied in hemophilia patients excluding patients with inhibitors. During the coagulation pathway, activated factor V (FVa) functions downstream as a cofactor of activated factor X (FXa) to amplify thrombin generation. We hypothesize that the expression of FVa via gene therapy can improve hemostasis of both factor IX and FVIII deficiencies, regardless of clotting factor inhibitor. A human FVa (hFVa) expression cassette was constructed, and AAV8 vectors encoding hFVa (AAV8/TTR-hFVa) were intravenously administrated into mice with hemophilia A and B with or without FVIII inhibitors. Hemostasis, including hFVa level, activated partial thromboplastin time (aPTT), tail clip, and the saphenous vein bleeding assay (SVBA), was evaluated. In hemophilia B mice, a dose of 4 × 10^13^ vg/kg AAV8/TTR-hFVa vectors achieved a complete phenotypic correction over 28 weeks. In hemophilia A mice, hemostasis improvement was also achieved, regardless of FVIII inhibitor development. *In vivo* hemostasis efficacy was confirmed by tail clip and SVBA. Interestingly, while minimal shortening of aPTT was observed at a lower dose of AAV8 vectors, hemostasis improvement was still achieved via *in vivo* bleeding assays. Collectively, FVa-based AAV gene therapy shows promise for hemostasis correction in hemophilia, regardless of inhibitor development and no potential risk for thrombosis.

## Introduction

Hemophilia is an inherited bleeding disorder caused by a deficiency of the functional clotting factor FVIII (hemophilia A) or FIX (hemophilia B). Current treatment by protein replacement therapy is constrained by the short half-life of the clotting factors, requiring repeated infusions at relatively large doses. The development of inhibitors (alloantibodies to clotting factors) remains the single most important obstacle for managing hemophilia with protein replacement therapy. These alloantibodies render replacement therapy ineffective, with an increase in the risk of serious bleeding, progressive arthropathy, and treatment-related costs.

Approximately 30–40% (1) of patients with hemophilia A and 5% of patients with hemophilia B can develop inhibitors after protein replacement therapy (2–4). In patients with inhibitors, the administration of clotting factors is ineffective, and poor control of hemorrhagic episodes mostly increases orthopedic complications. Treatment with immune tolerance induction (ITI) therapy is successful in about 70% of hemophilia A and 30% of hemophilia B (5, 6). In addition to ITI therapy, the bypass product FVIIa has also been applied in hemophilic patients with inhibitors (7, 8). Other novel options, including emicizumab, a bispecific mAb that bridges FIX and FX in the clotting cascade, has been a major addition for hemophilia A with or without inhibitors with some potential safety concerns (9–11).

Gene therapy could ultimately provide a cure and avoid the need for repeated clotting factor infusions. Among the gene therapy vectors, adeno-associated virus (AAV) vectors have been used successfully in numerous preclinical applications. AAV is a single-stranded DNA virus, while AAV vectors have been shown to induce a long-term and stable therapeutic gene expression over 10 to 15 years in dogs, primates, and human (12–14). AAV can infect both dividing and non-dividing cells. Although there is some evidence of AAV integration events in rodent and canine models as summarized in recent discussion of AAV Integration Roundtable meeting initiated by the American Society of Gene & Cell Therapy (15), the risk of integration has putatively been recognized as low. Due to extensive preclinical studies, over 150 clinical trials with AAV vectors are ongoing, and great success has been achieved in patients with diseases such as inherited blindness and hemophilia. In multiple clinical trials with hemophilia B patients, after systemic administration of AAV vectors into the liver, a stable therapeutic level of FIX activity (16, 17) and a supraphysiological level have been achieved (18). In patients with hemophilia A, liver targeting with a high dose of AAV vectors encoding human FVIII also supports FVIII expression in the blood from 4% to over 100% (16, 19, 20). However, this therapy is currently restricted to patients who are negative for FVIII or FIX inhibitors.

Clotting factor V (FV) is synthesized as a single-chain protein in the liver, which is composed of A1, A2, B, A3, C1, and C2 domains, in order. FV is a pro-cofactor and in this state does not have procoagulant activity. It is activated by thrombin via limited proteolysis to release the B domain, and the interaction of the HC and the LC generates the procoagulant heterodimer FVa (21). As a cofactor, FVa interacts with FXa to form a prothrombinase complex, which is essential for the rapid generation of thrombin (22). Indeed, FVa is able to enhance the rate of thrombin generation by approximately 10,000-fold (23). Therefore, treatment with FVa can theoretically bypass any upstream factors involved in the coagulation cascade, and most importantly, FVa function still remains under the regulation of activated protein C. Activated protein C is one of the principal physiological inhibitors of coagulation and degrades FVa, ameliorating the concern of thrombosis.

In this study, we explored the therapeutic potential of FVa delivered by AAV vectors in hemophilia mice with or without pre-existing FVIII inhibitors. The efficacy and safety with AAV vectors encoding human FVa as a bypass product were achieved in hemophilia mouse models.

## Materials and methods

### HEK293 cells and western blot

HEK293 cell lines were incubated at 37°C in 5% CO_2_ in Dulbecco’s modified Eagle medium (DMEM) supplemented with 10% fetal bovine serum (FBS) and penicillin–streptomycin. The cell supernatant 72 h after transfection was subjected to sodium dodecyl sulfate–polyacrylamide gel electrophoresis (SDS-PAGE), followed by transferring to a polyvinylidene difluoride (PVDF) membrane, and then primary antibodies of anti-hFVa (Santa Cruz Biotechnology, Dallas, TX, United States, and Haematologic Technologies, Essex Junction, VT, United States) and conjugated secondary antibody (Abcam, Cambridge, MA, United States) were added for protein detection.

### Construction of adeno-associated virus vectors and adeno-associated virus vector production

**FIGURE 1:**
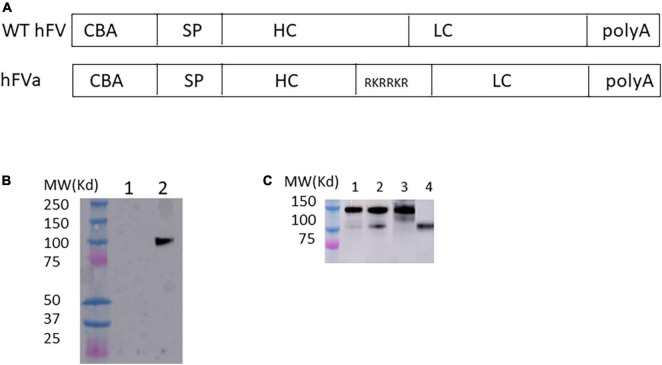
FVa formation in transfected HEK 293 cells. **(A)** Diagram of human FVa construct for *in vitro* expression in HEK 293 cells. CBA, chicken beta actin promoter; SP, signal peptide; HC, heavy chain; LC, light chain. **(B)** Plasmids of hFVa driven by CBA promoter were transfected in HEK 293 cells; 3 days later, the supernatant was harvested for detection of hFVa with the GMA-044 detection antibody, which specifically recognizes the hFV HC. Lane 1: green fluorescent protein (GFP); lane 2: hFVa. **(C)** Plasmids of full length FV (“hFV”) and BDD-hFVa (“hFVa”) were transfected in HEK293 cells; 3 days later, the supernatant was harvested. Thrombin at a final concentration of 10 U/ml was added to the supernatant; 10 and 30 min later, Western blot was performed using anti-hFVa as the detection antibody for light chain of FV. Lane 1: hFV + thrombin 10 min; Lane 2: hFV + thrombin 30 min; lane 3: hFV; lane 4: hFVa.

**FIGURE 2:**
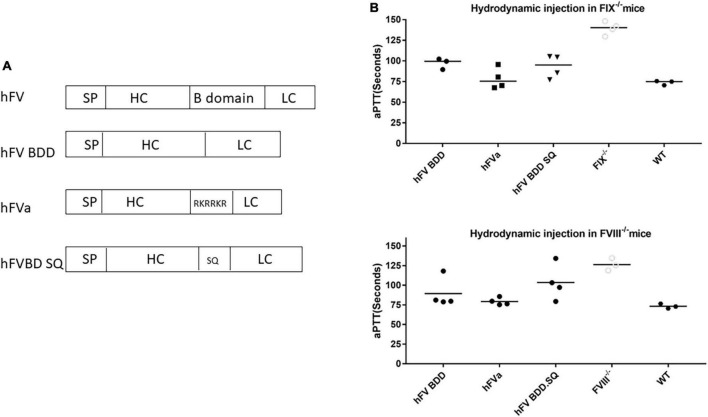
FVa was functional in hemophilia mice after hydrodynamic injection of hFVa plasmids. **(A)** Constructs of FVa “hFV BDD”: complete deletion of B domain; “hFVa”: complete deletion of the B domain and a furin cleavage site linker between the FV heavy chain (HC) and the light chain (LC); “hFVBD SQ”: complete deletion of the B domain with an SQ sequence inserted between the HC and the LC. **(B)** aPTT change after hydrodynamic injection. In both FIX^– /–^ and FVIII^– /–^ mice, the three plasmid constructs were tested, and FVIII/FIX-specific aPTT was measured 48 h after hydrodynamic injection. FIX^– /–^/FVIII^– /–^ mice (“FIX^– /–^”/“FVIII^– /–^”and their “WT”) controls injected with normal saline were used as the controls.

All human FV DNA sequences (Figures 1, 2) were synthesized by GenScript (Piscataway, NJ, United States) and driven by the chicken beta actin (CBA) promoter (Figure 1) or the liver-specific transthyretin (TTR) promoter (500 bp including an MVM intron) and fused with BGH polyA (230 bp), as described previously (24). These AAV FVa constructs (Figure 2) were cloned by either deleting the B domain (hFV BDD) or linking the heavy chain [HC, with signal peptide (SP), 737aa–2,211 bp] with light chain (LC, 651aa–1953 bp) using a furin cleavage motif (RKRRKR) (hFVa) or with an SQ sequence (SFRNPDNIAAWYLRRKR) (hFVBD SQ) (25). Both plasmids of full length and BDD-hFVa driven by the CBA promoter (Figure 1A) were transfected into HEK293 cells, with the supernatant subjected to the thrombin (Roche, Mannheim, Germany) cleavage. The supernatant from hFVa plasmid transfection was also tested for activated protein C (Sigma, St. Louis, MO, United States) inhibition assay based on FVIII-specific aPTT assay. All vectors were produced following the triple transfection protocol and tittered at the Virus Vector Core Facility at the University of North Carolina at Chapel Hill, as described previously (26).

### Animal care

FVIII^–/–^ (B6129S background) and FIX^–/–^ mice (C57B6 background) were initially supplied by Dr. H. H. Kazazian Jr (27) and Dr. D.W. Stafford (28), respectively, and were bred in-house. Male mice were used for all the gene therapy studies. Plasma samples were collected from the retro-orbital plexus and placed in 3.2% sodium citrate and stored at –80°C until analysis. Animal protocols were approved by the Institutional Animal Care and Use Committee of the University of North Carolina at Chapel Hill.

### Induction and quantification of anti-FVIII inhibitor

To remodel the pre-existing FVIII inhibitor development in hemophilia A mice, FVIII^–/–^ mice were treated with rhFVIII (Advate, Baxter, Westlake Village, CA, United States) at a dose of 100 IU/kg, once a week for 4 weeks. The titer of anti-human FVIII inhibitor was measured by using the Bethesda assay, as previously described, using a STart 4 Coagulation Analyzer (Diagnostica Stago, Parsippany, NJ, United States) (29).

### Hydrodynamic injection

Purified plasmids (100 μg/mouse) in 2 ml of phosphate-buffered saline were injected hydrodynamically into the lateral tail vein of mice within 5–8 s, as described previously (30). Plasma samples were harvested 48 h later for aPTT analysis. At least three mice were included for each treatment group.

### FIX- or FVIII-specific activated partial thromboplastin time and quantification of hFVa expression by enzyme linked immunosorbent assay (ELISA)

Given the presence of FV/FVa in the final common pathway of blood coagulation and either hemophilia A or B has a normal PT time, we adopted FVIII/FIX-specific aPTT as the parameter to demonstrate hemostasis improvement after gene therapy, along with tail clip and the saphenous vein bleeding assay (SVBA). FIX- or FVIII-specific aPTT tests were used to monitor the hemostasis improvement on the STart 4 coagulation analyzer by incubating 50 μl mouse plasma samples diluted in Owren-Koller buffer (Diagnostica Stago), 50 μl hFVIII- or FIX-deficient plasma (George King Bio-Medical, Overland Park, KS, United States), and 50 μl aPTT reagents for 3 min and then adding 50 μl 0.025 M calcium chloride. Pooled plasma from wild-type mice was used as the control.

For the quantification of hFVa expression in mouse plasma, we employed an ELISA method with coating and detection antibodies (Affinity Biologicals, Ancaster, ON, Canada). Activated human Factor V (Haematologic Technologies, Essex Junction, VT, United States) was used to construct the standard curve.

### Tail clip and the saphenous vein bleeding assay

The tail clip was performed as described (31) (*N* = 44 FVIII^–/–^ mice treated with AAV8.FVa). In brief, 3 mm of the distal tail was transected, and the proximal tail was placed in a pre-warmed and pre-weighed tube. After 40 min of the tail clip or upon death, blood loss per gram body weight was calculated.

Saphenous vein bleeding assay was performed on the right saphenous vein by piercing it with a 23-G needle (*N* = 23 FVIII^–/–^ mice treated with AAV8.FVa). Blood was gently wicked away until hemostasis occurred. The clot was then removed to restart bleeding, and the blood was again wicked away until hemostasis occurred again. The number of clots that occurred over the course of 30 min was recorded (32).

### Rotational thromboelastometry (ROTEM) and thrombin/antithrombin III complex detection

For fibrinolysis analysis, ROTEM was employed, which can provide global information on the dynamics of clot development, stabilization, and dissolution, which reflect *in vivo* hemostasis. Whole blood was collected from the inferior vena cava at killing in FIX^–/–^ mice treated with AAV8.FVa and placed in 3.2% sodium citrate mixed at a ratio of 9:1. Then, 300 μl of the resulting mixture was coagulated with 20 μl of 0.2?M CaCl_2_, and finally, 3 μl of tissue plasminogen activator (DiaPharma, Louisville, KY, United States) was added to anticoagulated whole blood to a final concentration of 1 ng/ml in a pre-warmed rotational thromboelastometer.

Thrombin–antithrombin (TAT) evaluation was carried out (*N* = 21 FIX^–/–^ mice treated with AAV8.FVa) from plasma collected as a terminal puncture of the inferior vena cava using an Enzygnost TAT micro ELISA system (Siemens Healthcare Diagnostics, Tarrytown, NY, United States).

### Statistical analysis

Results of a one-way, non-parametric analysis were analyzed using GraphPad Prism 6.0 software (GraphPad Software, La Jolla, CA, United States). An adjusted *P*-value < 0.05 was considered a statistically significant difference.

## Result

### Detection of factor V after transfection of factor V plasmid in HEK 293 cells

To test whether the FVa transgene construct can be properly processed intracellularly, the plasmid encoding human FVa (hFVa), driven by a CBA promoter (Figure 1A), was transfected into HEK 293 cells, as displayed in Figure 1B. The heavy chain (HC, 110 kd) was detected with the antibody GMA-044, which specifically recognizes the hFV HC. As shown in Figure 1C, FV cleavage was seen by the addition of thrombin to full-length FV plasmid-transfected supernatant (light chain detected, weak band 10 min later at lane 1, and a clear band 30 min later at lane 2, while no band without thrombin at lane 3). The aPTT was significantly prolonged after the addition of activated protein C to the supernatant from hFVa plasmid transfection (data not shown), implicating that hFVa was subjected to inhibition by activated protein C.

### Hydrodynamic injection of factor V transgene plasmids leads to activated partial thromboplastin time improvement in both hemophilia A and B mice

Next, we investigated whether the engineered sequence with SQ activation peptide sequence (33) could increase the expression efficiency. Given the efficiency and convenience of hydrodynamic delivery of genes of interest, we employed hydrodynamic injection to assess the efficacy of different FV constructs in the liver. As shown in Figure 2, compared to the construct with only B domain deleted FV versus FV constructs with the B domain deletion and replacement by the SQ linker or furin cleavage, the FVa plasmid with the furin cleavage domain showed a slight tendency to induce a better hemostasis improvement with a shorter aPTT in both hemophilia A and B mice. These results indicated that FVa protein can be properly processed and formed by the furin cleavage intracellularly. Therefore, “hFVa” was chosen as the candidate for AAV vector package and further *in vivo* study.

### Hemostasis improvement after factor V gene therapy in FIX^–/–^ mice

**FIGURE 3:**
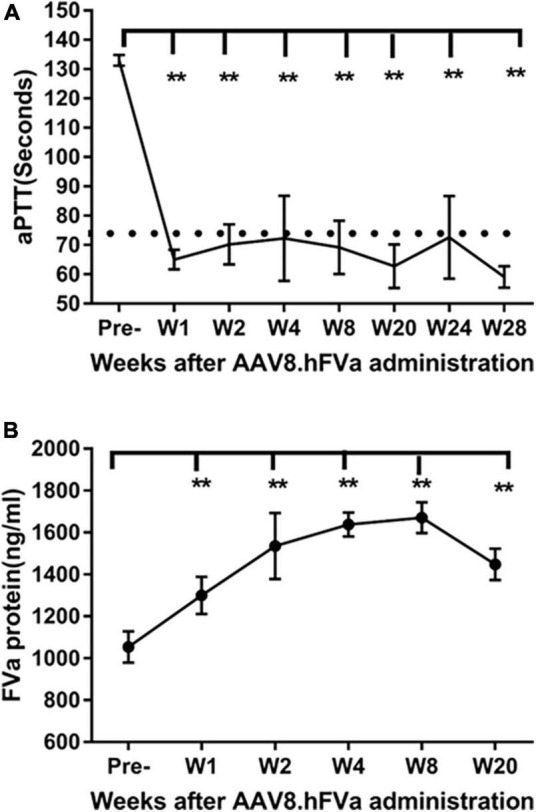
Complete phenotypic correction after administration of AAV8/hFVa in FIX^– /–^ mice. 4 × 10^13^ vg/kg body weight of AAV8/hFVa was administrated into FIX^– /–^ mice via the tail vein (*N* = 5, male, at ages 8–10 weeks). Blood was harvested for aPTT assay and hFVa quantification by ELISA. **(A)** aPTT assay after AAV8/hFVa administration, and dashed line represents normal WT mice. **(B)** hFVa concentration in blood was quantitated by ELISA. Data are presented as an average with standard errors. ** Compared with pre-treatment, *P* < 0.01 (two-way ANOVA with multiple comparisons and unpaired *t*-tests).

To study whether hFVa induces a phenotypic correction in a hemophilia setting, we constructed AAV8 vectors encoding hFVa driven by the TTR promoter (AAV8/TTR-hFVa). A dose of 4 × 10^13^ vg/kg of AAV8/TTR-hFVa was administrated into FIX^–/–^ mice via the tail vein (*N* = 4–5 mice/time point). Plasma was collected. aPTT was analyzed during a 28-week follow-up after AAV8/hFVa vector injection. The quantity of expressed hFVa was also measured by ELISA. As shown in Figure 3A, a complete phenotypic correction with a normal aPTT was achieved when compared to the wild-type mice (about 72 s of aPTT), indicating that AAV8/hFVa gene therapy improved hemostasis in hemophilia B mice. Due to the lack of sensitive and reliable assays to differentiate the human factor V and FVa, we adopted an ELISA method using the anti-human FV antibody as the capture and detection antibodies, and hFVa as the standard. With the baseline level of FV in FIX^–/–^ mice (Figure 3B) at around 1,000 ng/ml, the level of FVa after AAV8/hFVa treatment showed a steady increase during the follow-up period from week 1 to week 20, which was consistent with the result from the aPTT analysis.

### Phenotypic correction in FVIII^–/–^ mice treated with low doses of AAV8/TTR-hFVa vectors

Next, we studied whether the administration of AAV8/hFVa was also able to induce hemostasis improvement in FVIII^–/–^ mice. A dose of AAV8/TTR-hFVa vectors (8 × 10^12^ vg/kg and 1.6 × 10^13^ vg/kg) was given. After 12–20 weeks of follow-up, a slight reduction of aPTT was observed (Table 1). At the end of experiments, all mice were used for tail vein transection to evaluate the hemostatic improvement in AAV8/FVa treatment *in vivo*. As displayed in Table 1, the survival from tail clip was significantly improved in hemophilia A mice treated with AAV8/FVa vectors when compared to that in naive hemophilia mice (95 vs. 61%); only two mice died among the 44 treated mice. In contrast to the results from aPTT, the blood loss from tail clip was significantly decreased in mice treated with AAV8/hFVa vectors (8.7 ± 7.2 g/kg body weight vs. 34.5 ± 12.9 g/kg body weight in naive FVIII^–/–^, *P* < 0.01), approximately close to the findings with wild-type mice (4.9 ± 4.4 g/kg). In untreated hemophilia A mice, considerable blood loss was found (34.5 ± 12.9 g/kg). When we further divided mice into low-dose (8 × 10^12^vg/kg) and high-dose (1.6 × 10^13^vg/kg) groups, there was no statistical difference between the two dose groups for both aPTT and blood loss from tail clip. In the studies, even with no obvious improvement in hemostasis from the aPTT assays, the *in vivo* bleeding via tail clip was significantly improved in hemophilia A mice after the administration of AAV8/hFVa vectors at low doses.

**TABLE 1:** Phenotypic correction of tail clip in FVIII^–/–^ mice treated with AAV8/hFVa vectors.

Mice treatment	*N*	aPTT time (s) pre-treatment	aPTT time (s) before tail clip	Survived/Ratio (%)	Blood loss (g/kg body weight)
Naïve FVIII^–/–^	13	114 ± 6.9 (104–124)	NA	8 (61%)	34.5 ± 12.9
AAV8.FVa	44	113 ± 5.8 (102–132)	99.1 ± 10.0 (82–118)*	42 (95%)**	8.7 ± 7.2**
WT	7	NA	NA	7 (100%)	4.9 ± 4.4

* Compared with naive FVIII^–/–^ aPTT of pre-treatment. ** Compared with naive FVIII^–/–^, *P* < 0.01.

### No over-correction of hemostasis with a high dose of AAV8/hFVa vectors in hemophilia A mice

**FIGURE 4:**
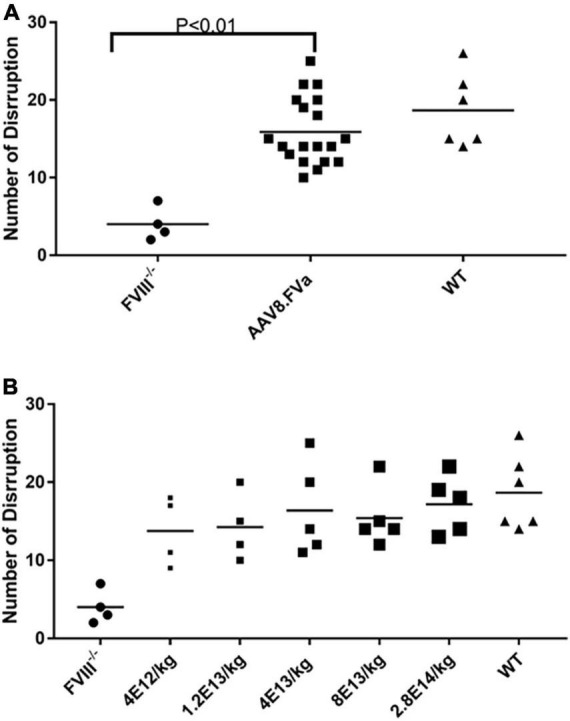
*In vivo* hemostasis improvement by saphenous vein bleeding assay (SABV) in hemophilia A mice treated with AAV8/hFVa. Five different doses of AAV8/hFVa vectors, from 4 × 10^12^ to 2.8 × 10^14^ vg/kg (*N* = 19 for AAV8/hFVa treated), were given to FVIII^– /–^ mice. At 12–20 weeks after AAV8/hFVa administration, SVBA was performed. The number of disruptions within 30 min was recorded. **(A)** A number of disruptions from five different doses of AAV8/hFVa were pooled together. **(B)** Number of disruptions from individual groups with different doses of AAV8/hFVa (*N* = 4–5/group).

Next, we studied the effect of AAV8/hFVa at different doses on hemostasis improvement in hemophilia A mice. To explore the potential minimal dose for hemostasis improvement and whether there was any dose-dependent potential hepatic toxicity, five different doses were used, from 4 × 10^12^ vg/kg to 2.8 × 10^14^ vg/kg. Other than aPTT analysis, SVBA was also performed 8–12 weeks after gene therapy treatment to evaluate the *in vivo* phenotypic correction. None of the mice showed any potential liver toxicity reflected by liver enzymes (data not shown). As displayed in Figure 4 and Table 2, four trends were drawn: (1) aPTT can be shortened in an approximately dose–response pattern. (2) *In vivo* bleeding assay with SVBA did not show dose-dependent hemostasis improvement. The disruption number was 18.7 ± 4.4 for the wild-type mice and 4.0 ± 1.9 for the untreated hemophilia A mice. The disruption number was over 10 for any mice treated with AAV8/hFVa vectors, regardless of the doses. There was no significant difference in the disruption number between any groups of mice receiving various doses of AAV8/hFVa vectors (*P* < 0.05). Pooled data from mice treated with five different doses of AAV8/hFVa vectors showed no difference in the disruption number compared to the WT mice (*P* > 0.05), consistent with data from the blood loss assay from tail clip (Table 1). (3) It is also worth noting that the aPTT was barely improved in mice with the lowest dose (4 × 10^12^vg/kg) of vectors, but the *in vivo* improvement in hemostasis via SBVA remained significant (disruption number of 13.7 ± 3.8). (4) When the highest dose of AAV8/hFVa (2.8 × 10^14^ vg/kg) was analyzed, the aPTTs were slightly shortened when compared to the dose at 8 × 10^13^ vg/kg (80.5–87 s vs. 86–102 s), but no significance was reached. This result indicated that over-correction of hemostasis was not induced even when extra high doses of AAV8/hFVa are used.

**TABLE 2:** Phenotypic correction by saphenous vein bleeding assay in FVIII^–/–^ mice treated with different doses of AAV8/hFVa vectors.

Mice treatment	*N*	aPTT time (s) pre-treatment	aPTT time (s) before tail clip	Number of disruptions
Naïve FVIII^–/–^	4	113 ± 5.0 (108–120)	NA	4.0 ± 1.8
AAV8.FVa	23	115 ± 5.3 (102–132)	99.1 ± 10.0 (82–118)	15.2 ± 4.1**
4 × 10^12^/kg	4	117 ± 5.2 (109–123)	115 ± 4.6 (107–119)	13.7 ± 3.8**
1.2 × 10^13^/kg	4	111 ± 5.5 (114–132)	105 ± 5.9 (97–112)	14.2 ± 3.7**
4 × 10^13^/kg	5	112 ± 2.0 (105–121)	106 ± 5.5 (95–112)	16.4 ± 5.3**
8 × 10^13^/kg	5	115 ± 3.7 (109–123)	92 ± 5.9 (86–102)	15.4 ± 3.4**
2.8 × 10^14^/kg	5	121 ± 1.6 (119–123)	83 ± 2.7 (80.5–87)	17.2 ± 3.3**
WT	5	NA	NA	18.6 ± 4.1

** Compared with naive FVIII^–/–^, *P* < 0.01. *P* > 0.05 between all treatment groups/WT.

### Factor V gene therapy improved hemostasis in FVIII^–/–^ mice with pre-existing anti-FVIII inhibitor

**FIGURE 5:**
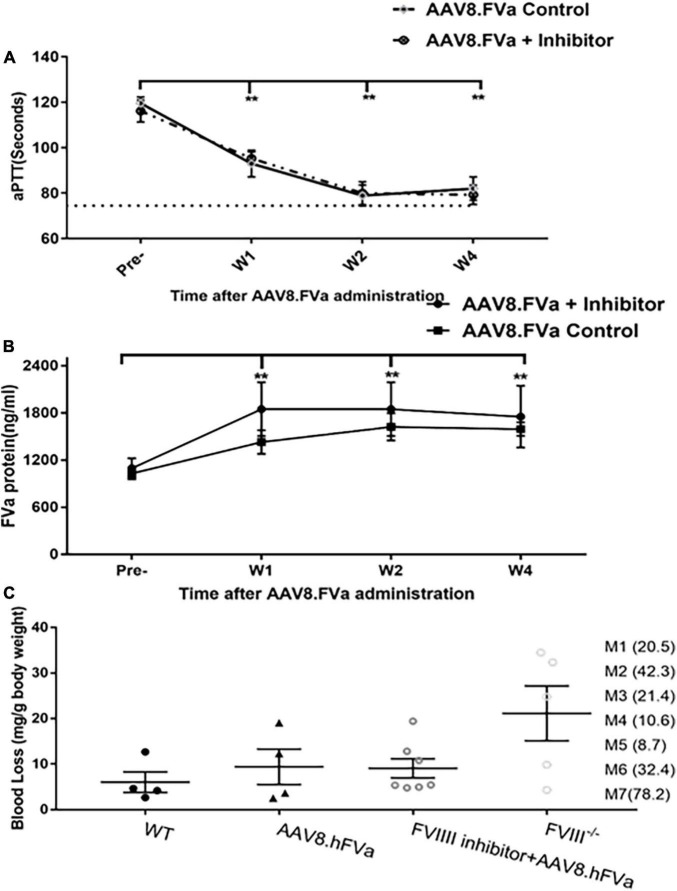
Administration of AAV8/hFVa vectors improved hemostasis in hemophilia A mice with inhibitors. **(A)** FVIII^– /–^ mice were first induced to develop anti-FVIII inhibitor by repeated FVIII protein infusion (“AAV8/hFVa + inhibitor,” *N* = 7). Hemophilia A without FVIII inhibitor was utilized as the controls (“AAV8/hFVa control,” *N* = 4). AAV8/hFVa vectors were administrated via the tail vein. Plasma was collected for aPTT assay at different time points. ** Compared with pre-treatment, *P* < 0.01 (two-way ANOVA with multiple comparisons and unpaired *t*-tests). **(B)** Plasma from the same mice (“A”) was measured for hFVa by ELISA. *N* = 6 for “AAV8/hFVa + inhibitor”; *N* = 4 for “AAV8/hFVa control.” ** Compared with pre-treatment, *P* < 0.01 (two-way ANOVA with multiple comparisons and unpaired *t*-tests). **(C)** Eight weeks post-AAV8/hFV injection, FVIII^– /–^ mice were subjected to the tail clip bleeding challenge. The blood loss was recorded. M1-M7 represent anti-FVIII inhibitor titer (BU/ml) of individual mouse. All data are presented as averages with standard errors.

In the aforementioned experiments, we have demonstrated that administration of AAV8/hFVa induced hemostasis improvement in both hemophilia A and hemophilia B mice without inhibitors. Next, we studied whether the phenotypic correction in hemophilia mice without inhibitors from the application of AAV/hFVa could also apply to hemophilia mice with inhibitors. FVIII inhibitors were first induced by administration of protein in hemophilia A mice. All the mice developed high titers of anti-FVIII inhibitor (from 8.7 to 42.3 BU/ml as shown in Figure 5C). At 1 week after the final FVIII inhibitor titer was quantified, AAV8/hFVa vectors were administrated at a dose of 8 × 10^13^ vg/kg. Hemostasis correction was assessed by measuring aPTT and the levels of FVa by ELISA from plasma collected at week 1 and week 4 post-AAV8/hFVa injection. As shown in Figure 5A, hemophilia A mice with FVIII inhibitors showed continuous shortening of aPTT clotting time after the administration of AAV8/hFVa vectors, similar to aPTT in mice without FVIII inhibitors. The shortening of clotting time coincided with the elevation of hFVa, as shown in Figure 5B.

At week 8 post-AAV8/hFVa vector administration, the mice were subjected to the tail clip bleeding challenge. As shown in Figure 5C, all seven mice treated with AAV/FVa gene therapy survived the tail clip, and the blood loss was significantly lower than that in naive hemophilia A mice. There was no difference of blood loss in mice with or without pre-existing anti-FVIII inhibitors after treatment with AAV8/hFVa vectors.

Overall, there were no statistical difference between mice with or without pre-existing FVIII inhibitor, implicating that FVa-based gene therapy can improve hemostasis in FVIII^–/–^ mice, regardless of pre-existing anti-FVIII inhibitor.

### No thrombotic risk was detected in hemophilic B mice treated with AAV8/hFVa

As no mice showed liver enzyme elevation, including the highest dose (2.8 × 10^14^/kg)-treated mice in the report (data not shown), the concern of thrombosis due to inappropriate activation of coagulation from continuous secretion of FVa after AAV gene delivery should be addressed, given that FVa participates in the common pathway of the coagulation cascade. Then two parameters were used to evaluate the risk of thrombosis following FVa-based gene therapy: TAT assay and fibrinolysis analysis on ROTEM.

**FIGURE 6:**
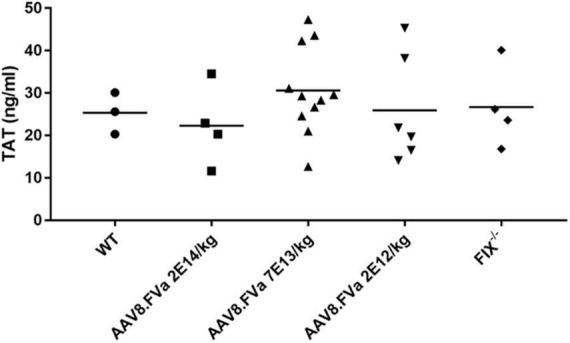
Evaluate the risk of thrombosis after AAV8/hFVa treatment by detection of the TAT complexes. TAT complexes were measured via ELISA from platelet-poor citrated plasma collected as a terminal puncture of the inferior vena cava at 20 weeks after AAV8/hFVa vector administration when the FIX^– /– –^ mice were killed. Age-matched WT and untreated hemophilia B mice served as the controls.

FIX^–/–^ mice were administrated AAV8/hFVa at the following doses: 2 × 10^12^/kg, 7 × 10^13^/kg, and 2 × 10^14^vg/kg. At 16–20 weeks after gene therapy, plasma TAT complexes were measured. As displayed in Figure 6, no elevation of TAT levels was observed in mice treated with AAV8/hFVa vectors even at the highest dose of 2 × 10^14^ vg/kg when compared with age- and gender-matched WT and untreated hemophilia B mice.

**FIGURE 7:**
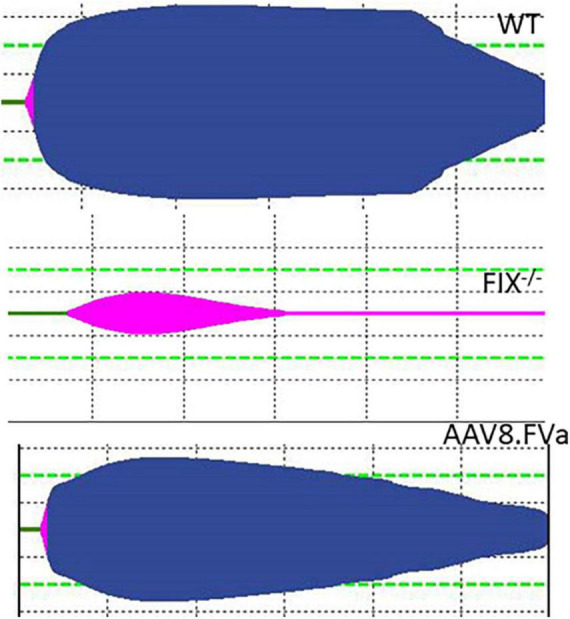
Evaluate the risk of thrombosis after AAV8/hFVa treatment by fibrinolysis on ROTEM. Fibrinolysis analysis using whole blood collected from the inferior vena cava at the end of experiments was performed on ROTEM for mice (Figure 6).

To further assess whether the normal fibrinolysis pathway was affected by persistent production of FVa, a ROTEM assay was conducted. As shown in Figure 7, the clot was dissolved completely in WT mice. By contrast, clot formation was barely developed in the FIX^–/–^ mice. However, the clot was formed in the FIX^–/–^ mice treated with AAV8/hFVa vector (at week 20) and then fully dissolved, a pattern similar to that in the WT mice. The result implicates that continuous expression of FVa does not lead to clot resistance with no thrombotic risk.

## Discussion

Adeno-associated virus vectors encoding the bypass molecule FVIIa have been used in hemophilia animal models with inhibitors, yet only partial improvement in hemostasis was achieved (34, 35). To overcome the issues of inhibitors and poor efficacy in a single-dose format, FVa-based gene therapy may represent a potentially safe and effective single-dose drug for treatment in hemophilia patients with or without inhibitors.

In this study, we found that FVa protein can be properly processed and formed by furin cleavage intracellularly. In hemophilia B mice, complete phenotypic correction was achieved over 28 weeks of follow-up within a normal activated partial thromboplastin time (aPTT). In hemophilia A mice with or without pre-existing FVIII inhibitors, hemostasis correction, represented by aPTT, was achieved. The phenotypic correction was also supported by tail clip and SVBA, even without improvement from the aPTT assay at low doses of AAV8/hFVa vectors. In comparison to WT and untreated hemophilia mice, gene therapy with AAV8/TTR-hFVa did not induce increased risk of thrombosis based on normal TAT levels and fibrinolysis on ROTEM. These results imply that continuous expression of FVa via AAV gene delivery would not lead to uncontrolled clot formation.

Using the AAV vector to deliver FVa for long-term expression allowed us to assess the risk of thrombotic formation related to FVa treatment. Based on the data from TAT detection and fibrinolysis analysis, no thrombotic events were observed in mice treated with AAV8/hFVa. The lack of thrombotic risk may be attributable to tight regulation of FVa by normal physiological mechanisms. Once circulating FVa blood levels are sufficient to complex with FXa for sufficient thrombin generation and phenotypic correction after administration of AAV vectors, the thrombin will activate protein C for rapid degradation of extra FVa to maintain a certain amount of FVa for thrombin production.

Given the unique features of AAV, including broad tropism, low immunogenicity, non-pathogenicity, and rare integration, AAV-mediated gene therapy has emerged as the proven platform for the treatment of multiple diseases. The efficacy and long-term expression of the transgene (FVIII/FIX) has been achieved in ongoing clinical trials for both hemophilia A and B (36). In this report, a relatively large dose of AAV8/hFVa vector was required to normalize the hemostasis correction in both hemophilia A and B mice. The size of FVa cDNA is approximately 4,200 bp. Due to size limitations for encapsulation in the AAV vector (37, 38), it is not possible to use a strong liver promoter of significant size to increase FVa expression. Future work should explore shorter strong liver-specific promoters, genetic optimization of FVa DNA sequences, or transduction enhancement through use of a customized capsids with stronger liver tropism. These enhancements would further reduce the potential for high-dose AAV vector-induced liver toxicity (39). In this study, FVa expression cassette (including the TTR promoter with an MVM intron 500 bp and hFVa 4,182 bp and BGH polyA 230 bp) is about 4,900 bp long. This total length of AAV FVa construct including two ITRs is less than 5.2 kb, which is maximum package limitation for AAV vector production (38). Actually, we performed alkaline gel electrophoresis and found that the intact genome was packaged (data not shown). The larger size of AAV construct is potential to induce truncate AAV genome packaged or produce more empty virions. Several approaches have been explored to use AAV vectors for large transgene delivery involving split vectors using splicing intron or intein (14, 15, 34, 35, 37, 39–44).

It is interesting to note that phenotypic correction was still achieved when tail clip/SVBA was performed in hemophilia mice even without improved aPTT after gene therapy. It is unclear why the data from aPTT analysis cannot predict the hemostasis correction *in vivo* after AAV/hFVa gene therapy in hemophilia mice. It is possible that the secreted FVa may play a different role under conditions of active hemorrhage (tail clip/saphenous vein bleeding in this study) or without active bleeding. Under the normal conditions without active bleeding, the “extra” FVa may be instantly inactivated after secretion, while under the condition of active bleeding, the circulating FVa quickly interacts with the trace amount of FXa to initiate the downstream of coagulation cascade. Whether potential platelet expression of FVa at the lower vector doses can be attributed to the *in vivo* hemostasis improvement warrants further investigation. Due to the inability of aPTT/PT to predict hemostasis improvement *in vivo* after FVa gene therapy, exploration of other measurements, for example, annual bleeding rate in a large animal model, should be taken into consideration in future clinical studies (11).

Several decades of innovation have produced novel, non-traditional molecular medicines that have greatly improved the clinical management of hemophilia. Some of these treatments include biphasic antibodies bridging FIX and FX to bypass the FVIII requirement, antibodies against tissue factor pathway inhibitor (TFPI), and antithrombin-specific small interfering RNA (siRNA) targeting natural anticoagulant pathways to “rebalance” hemostasis (45). However, the longer half-lives of the antibody products or out of the loop of physiological coagulation regulation of these therapies has led to reports of thrombosis or associated events. Gene therapy, especially with AAV vectors, still holds the promise of a one-time infusion cure for hemophilia. Nonetheless, thrombotic events and concerns with overexpression of clotting factors have been reported or brought to notice in ongoing clinical trials of both hemophilia A (NCT04370054) and hemophilia B (NCT03369444).

Both FV and factor X (FX), as well as their activated forms FVa and FXa, are involved in the common pathway of the coagulation cascade. In addition to FVa, activated factor X (FXa) has also been considered a potential target for hemophilia therapy (46, 47). An ongoing phase 1b clinical trial of FXa has been carried out in patients with intracerebral hemorrhage and has confirmed its safety profile. FXa is rapidly inactivated by circulating protease inhibitors, resulting in a short half-life (<1–2 min). Furthermore, FXa can activate a range of procoagulant clotting factors, possibly leading to pathological activation of coagulation and is closely related to vascular disorders. Therefore, we have chosen its cofactor, FVa, as the candidate to treat hemophilia via a gene delivery approach. Although treatment with FVa has been proposed as a protein therapy in animal models in hemophilia, due to its very short half-life, the thrombosis risk with FVa therapy has not been fully evaluated. Administration of AAV8/hFVa leads to circulating FVa blood levels sufficient to complex with FXa produced in response to injury, resulting in adequate thrombin production and phenotypic correction. Thrombin then activates protein C for rapid degradation of the remaining circulating FVa, halting the clotting process. Constant endogenous liver production of FVa rebuilds circulating levels available to complex again with FXa upon the next injury. This theory is supported by our dose–response studies. Increasing the vector dose 70-fold dramatically shortened the aPTT, but the *in vivo* bleeding phenotype was corrected without the same magnitude of change. In combination with TAT data and fibrinolysis analysis via ROTEM, all of these results support the safety profile with AAV vector-mediated delivery of FVa in hemophilia with a minimal concern of thrombotic risk.

The short half-life of hFVa restricts its application in clinical trials. Recently, FVa mutants by engineering FVa APC cleavage sites have been proposed as a novel approach to bypass inhibitors (48–50) with a good safety profile including risk of thrombosis in the lung and immunogenicity in mice (50). However, these FVa mutants may not be good candidates for gene therapy due to evidence that mutated FVa is out of regulation of normal physiological hemostasis mechanisms and potential thrombosis risk. Gene delivery of wtFVa with AAV vectors has several advantages over the replacement treatment with mutated FVa protein: (1) AAV vectors have been successfully applied in patients with hemophilia A and B and proven to be safe. (2) Only one infusion is required since long-term transgene expression has been observed in preclinical animal models and human clinical trials. (3) There is no contamination from the processes required for protein production and purification. (4) There is no need for an extra step to cleave FV using thrombin to generate FVa. (5) The wtFVa will be directly formed after expression from AAV gene therapy. (6) Its function will be closely regulated by normal physiological mechanisms, which is considered to minimize the potential risk of thrombosis from high expression of FVa, if any, as evidenced in our study. Therefore, the AAV/FVa technology raises the promise for broader application for hemophilia management in patients with inhibitors or without inhibitors. However, a relatively higher dose of AAV doses is required to achieve hemostasis correction. Further optimization of the expression cassette, for example, choosing stronger promoters and transgene optimization, may be necessary for translational success. Given the similar molecular structure between FV/FVa and FVIII, the potential immunological risk of FV/FVa *per se* may also require further characterization.

In conclusion, FVa-based AAV gene therapy showed high promise for hemostasis correction in hemophilia, regardless of clotting factor inhibitor development and without risk of thrombosis. The approach has the potential to achieve the goal of “one drug for multiple clotting factor deficiencies” in patients with hemophilia.

## Data availability statement

The original contributions presented in this study are included in the article/supplementary material, further inquiries can be directed to the corresponding authors.

## Ethics statement

The animal study was reviewed and approved by the University of North Carolina at Chapel Hill.

## Author contributions

JS, XC, ZC, and AD: contribute to study design and acquisition of *in vivo* and *in vitro* results. TN: statistical analysis. JS, TN, and CL: overall study conception and design, and interpreted data. All authors were involved in drafting the manuscript and gave approval of the manuscript.
